# Childhood trauma, emotions, and the eating disorder voice

**DOI:** 10.1186/s40337-026-01650-6

**Published:** 2026-06-01

**Authors:** Kirsty Matthews, Marc Williams, Falguni Nathwani, John Fox

**Affiliations:** 1 South Wales Doctoral Programme in Clinical Psychology, Cardiff University, Cardiff, Wales, UK; 2 University of Sheffield Doctorate in Clinical Psychology Programme, Sheffield, England, UK

**Keywords:** Eating disorders, Eating disorder voice, Trauma, Adverse childhood experiences, Emotional difficulties, Alexithymia, Emotion regulation, Beliefs about emotion

## Abstract

**Background:**

Many individuals with an eating disorder report experience of an internal ‘voice’ which represents their disorder. However, research exploring the aetiology of this phenomenon is still limited. Past research has identified associations between adverse childhood experiences, emotional difficulties and eating pathology. Recent qualitative evidence suggests that the ‘voice’ specifically may be of relevance in this model; developing during difficult times in an individual’s life and offering a sense of safety and guidance amidst adversity and associated emotional difficulties. The present study aimed to examine the relationship between childhood adversity, emotions, and the eating disorder voice experience.

**Method:**

In total, 148 participants (recruited via social media and Cardiff University) completed self-report measures exploring childhood trauma, beliefs about emotions, difficulties with understanding, expressing, and regulating emotions, eating disorder voice appraisals (specifically, perceived power of the voice), and eating disorder pathology. All participants had self-reported experience of an eating disorder voice, but the sample varied in terms of diagnoses and presentations.

**Results:**

The relative power of the eating disorder voice was found to be significantly positively associated with childhood emotional abuse (but no other forms of abuse), and this relationship was partially mediated by difficulties with emotion regulation. Greater voice power was significantly positively correlated with eating disorder pathology.

**Conclusions:**

These findings provide preliminary evidence of associations between childhood experiences, emotion regulation difficulties, and the eating disorder voice. Further research seeking to elucidate this complex phenomenon is recommended. Limitations, future research directions and clinical implications are discussed.

## Abstract (plain language summary)

Some people with eating disorders describe experiencing an internal “eatingdisorder voice.” This voice may criticise, control, comfort, or guide the person, and can feel powerful and difficult to ignore. Although thisexperience is commonly reported, researchers still know relatively little about how it develops or why it becomes influential.Previous research has shown that difficult childhood experiences and problems with managing emotions are linked to eating disorders. Recent qualitative studies have also suggested that the eating disorder voice may emerge during stressful or painful periods in life, sometimes appearing to offer safety,structure, or emotional protection. This study explored whether childhood adversity and emotional difficulties were associated with how powerful the eating disorder voice feels. A total of 148 participants with experience of an eating disorder voice completed online questionnaires about childhood trauma, emotional experiences, emotion regulation difficulties, eating disorder symptoms, and the perceived power of theeating disorder voice. The findings showed that people who reported higher levels of childhood emotional abuse were more likely to experiencethe eating disorder voice as powerful. Difficulties regulating emotions partly explained this relationship. The study also found that a more powerful eating disorder voice was linked to more severe eating disorder symptoms. These findings suggest that emotional abuse and difficulties managing emotions may play a role in the development or maintenance of the eating disorder voice. The results highlight the importance of considering emotional experiences and childhood adversity when supporting individuals who experience an eating disorder voice. Further research is needed to better understand this complex experience and how treatment approaches might address it.

## Introduction

Eating disorders are psychiatric disorders characterised by maladaptive eating patterns and habits. The term ‘eating disorder’ incorporates several diagnoses under the DSM-5 [1]; Anorexia Nervosa (AN), Bulimia Nervosa (BN), Binge-Eating Disorder (BED), Otherwise Specified Feeding and Eating Disorder (OSFED) and Avoidant and Restrictive Food Intake Disorder (ARFID). Eating disorders are notoriously challenging to effectively treat, with high relapse rates and underwhelming treatment outcomes for many [2–4]. The compounding physiological aspects of the disorder create further complexity, as risk is often high; indeed, Anorexia Nervosa has the highest mortality rate of any mental health disorder [4]. While many can and do recover from eating disorders with appropriate support, psychological interventions available continue to show poorer long-term outcomes, and currently no therapeutic approach has been identified as a frontrunner in the treatment of eating disorders [2, 3, 5]. This could perhaps be because the underlying mechanisms of eating disorders are still not well understood [6]. Such underlying mechanisms could include, for example, the frequently reported experience of a voice in eating disorders [7, 8].

Individuals with eating disorders often report experiencing a voice, commonly described as an internal commentary, which focuses on weight, shape, and eating and how they relate to self-worth [7]. This phenomenon is also often referred to as the “Anorexic Voice”, although research indicates that the voice is often present across eating disorder types [9, 10]; thus, within the current paper, this phenomenon will be referred to as the ‘Eating Disorder Voice’ (EDV). How the EDV is experienced can vary; while a majority of individuals perceive the EDV as internal and a reflection of their own thoughts and beliefs, some report experiencing the voice as external and entirely separate to the self [11]. It is likely the voice experience exists on a spectrum, from entirely one’s own thoughts, to an entirely external voice [8].

Qualitative explorations of people’s experiences of the EDV [12, 13] suggest that the voice tends to emerge in the early stages of the illness, often during a time of vulnerability in the individual’s life. It can present as a comfort, a solution, a distraction from problems, and a companion and guide. However, the voice can become critical and dominating over time, punishing and degrading if the individual fails to meet the increasingly high expectations of the voice. Some report the voice can mimic the patterns of bullies and abusers in their lives [13, 14]. Often, individuals describe striving to avoid the voice’s wrath by submitting to its will [8]. It is unclear why this change in the voice occurs, but perhaps is representative of the ambivalent nature of people with EDs in relation to their disorder [15].

Research and reports from service-users and clinicians thus far indicate that the EDV is a common experience; a systematic review [15] exploring the eating disorder voice experience found that over 90% of participants with an eating disorder described a voice experience (significantly higher than healthy controls). Noordenbos and colleagues [9] found in their quantitative study all participants who had experience of an EDV reported hearing the voice at least once a week, with some reporting a near-constant voice (again, significantly more than healthy controls who reported a voice). Another study [16] found similarly high rates of the EDV experience. Other research [17] exploring the relationship between the EDV and eating disorder pathology in people with AN reported a significant association between negative eating attitudes and greater perceived voice power, while the combined characteristics of powerful and malevolent voices were associated with lower BMI. In addition, in a clinical sample of women diagnosed with AN [18], characteristics of the voice were found to be associated with severity of ED pathology; for example, perceived voice benevolence was associated with more disordered eating attitudes, and longer duration of ED was significantly positively associated with perceived voice omnipotence. A qualitative analysis [19] also found that addressing the EDV through the use of voice dialogue techniques within therapy was of great benefit and increased motivation and optimism for change; these findings highlight the potential value of addressing the EDV in the treatment of EDs. Given the prevalence and significance of the voice experience within EDs, improving understanding of the voice could be of importance in guiding interventions for eating disorders; yet research exploring this complex phenomenon is limited [8]. The origins and influences of the EDV remain unclear; however, the literature suggests childhood experiences may play an important role [10, 14].

Research has established clear links between adverse and traumatic childhood experiences and eating disorder pathology more broadly [20]. For example, one meta-analysis [21] found a significant association between childhood abuse (sexual, physical, and emotional) and eating disorders. Their findings suggest a significant relationship between abuse and EDs overall: those who experienced childhood trauma were over three times more likely to have an ED than those without trauma. With regards to specific ED subtypes, BED and BN were significantly associated with all types of abuse, whereas AN was only significantly associated with physical abuse. However, a later meta-analysis [22], which included additional papers, found significant associations between AN and emotional abuse as well, and a significant association between the AN binge-purge subtype (but not the restricting subtype) and sexual abuse was also identified.

Despite research indicating eating disorder pathology more broadly is associated with childhood trauma, there is yet limited research exploring how childhood trauma relates specifically to the EDV. There are many potential mechanisms explaining the link between childhood trauma and voice-hearing in adulthood, including the impact of self-evaluations, depression, and anxiety [23]; it is possible that this also applies to the EDV. Another study [24] found evidence for the indirect effect of childhood adversity on psychosis (including hearing voices) via higher levels of dialogic inner speech (i.e., a back-and-forth dialogue with oneself). One study thus far has investigated the relationship between trauma and the EDV specifically [10]. The authors focused on dissociation as the mechanism through which trauma influenced the EDV; only emotional abuse was significantly associated with power of the EDV, and their findings identified dissociation as a partial mediator in this relationship. The authors recommended that studies investigate further potential mediational factors between childhood trauma and the EDV.

One potential mediator of interest is underlying emotional factors. Specifically, the present study is interested in three related but distinct emotion-related mechanisms: the roles of alexithymia (difficulty experiencing, identifying, and expressing emotions), beliefs about emotions, and emotion regulation difficulties.

Several researchers have sought to elucidate the mediating role of emotions in the association between adverse childhood experiences and ED pathology more generally. Two papers [25, 26] discuss the role of emotional invalidation in development of ED pathology, using Linehan’s [27] Dialectical-Behavioural Therapy (DBT) model. The DBT model [27] outlines the role of environments that invalidate and discourage emotional expression (for example, ones that punish displays of anger, fear, or sadness, and promote suppression of emotions) in the development of beliefs that emotions are dangerous or bad and should not be felt. These beliefs can be triggered when an emotion deemed unacceptable is experienced, and results in a secondary emotional response (for example, guilt at feeling angry, anger at themselves for feeling sadness). Corstorphine [25] further expands on this and posits that, for some, these beliefs and associated secondary emotional responses can result in significant emotional distress and confusion, and lead to individuals engaging in maladaptive emotion regulation strategies, such as ED behaviours to block and suppress emotional experiences and expression. Suppression, however, often has the unintended consequence of making emotions more distressing and confusing, creating a vicious cycle. Individuals living in emotionally invalidating environments are less likely to learn more adaptive ways of regulating emotions, further exacerbating difficulties [25].

The three emotional difficulties relevant to the current study (alexithymia, emotion regulation and negative beliefs about emotions) all appear particularly relevant to this population in the wider research into eating disorder pathology. Firstly, research has shown strong links between eating disorder pathology and emotion regulation, with greater difficulties in emotion regulation associated with more severe ED pathology [28]. A meta-analysis [29] of emotion generation and regulation in AN identified key difficulties with emotion regulation in comparison to healthy controls, with greater reliance on maladaptive emotion regulation strategies observed in participants with AN. Oldershaw and colleagues [30] posit that the disorder emerges as a method of regulating emotion in the context of pre-existing difficulties with emotion (linked with childhood experiences) which have caused confusion, poor integration of cognitive, affective and physiological aspects of emotion, and overwhelm in the face of emotional experiences. Such difficulties are not unique to individuals with AN. One study [31] reported women across all ED subgroups were more likely than healthy controls to report suppression of emotion (a maladaptive emotion regulation strategy) and less likely to report use of more adaptive emotion regulation strategies. Another paper [32] similarly reported emotion regulation difficulties and maladaptive regulation strategies across eating disorder diagnoses and reported improvements in emotion regulation post-treatment across ED subgroups.

The findings of a review [33] provide support for the mediating role of emotion development between childhood trauma and ED pathology. They found significant associations between childhood trauma and eating disorders and identified a variety of mediating factors, including dissociation, alexithymia, emotion dysregulation, anxiety, and depression. The authors suggest eating disorders could have a variety of functions, including suppressing difficult emotions resulting from trauma. Another study [34] found indirect, significant relationships between childhood emotional abuse and AN, with emotion regulation difficulties identified as a significant mediator of this relationship, providing further evidence for the mediating role of emotions. A survey [35] of participants with ED diagnoses (across all types) reported higher levels of emotional intensity, less acceptance of emotions, less emotional awareness and clarity, and more emotion regulation difficulties; with greater reliance on maladaptive regulation strategies and less use of adaptive emotion regulation strategies in comparison to healthy controls. These findings provide further evidence for the potential importance of emotions (and specifically, alexithymia, beliefs about emotions, and emotion regulation difficulties) as mediators for the relationship between childhood trauma and general ED pathology.

While the mediating roles of different facets of emotion-related difficulties have been examined in relation to ED pathology, there is yet little understanding of how emotions may be of importance in the relationship between childhood trauma and the EDV, specifically. One qualitative, grounded theory paper thus far has explored this [14], using interviews to explore the EDV, emotions, and childhood experiences in individuals with AN. Their paper outlined how adverse childhood experiences led to individuals feeling unsure and unsafe in their relationships with others, and subsequently resulted in unsafe, confusing, and overwhelming internal worlds. Many described difficulties with emotional regulation, feeling out of control and overwhelmed by emotion. Participants reported that the voice offered safety within this context of relational and internal unsafety, developing as a “guiding light”, helping them to become a better person, and offering comfort. However, this safety was deemed conditional, with voices becoming cruel and abusive when rules were not followed. The EDV was compared to grooming experiences, with the voice using strategies such as isolating the individual, exploiting vulnerabilities, and using threats and bribes to ensure the individual remained reliant and compliant. This paper indicates the potential mediating role of emotional difficulties in the relationship between childhood trauma and the EDV, with the EDV providing support to manage the distress of childhood trauma. It also suggests that the voice experience could be of significance in the broader relationship between traumatic experiences, emotions, and ED pathology. The grounded theory established in this paper [14] provides a unique model to make sense of how the EDV can develop during periods of relational unsafety and associated internal unsafety (for example overwhelming emotions and difficulty with managing and understanding these) and provides a sense of (conditional) safety.

While Morrison and colleagues [14] provided rich, in-depth accounts, a quantitative examination of the relationships between emotions, childhood trauma, and the EDV could be of benefit, to expand understanding and explore the relevance of this model in a larger sample. The present study seeks to investigate the model posed further, using quantitative methods. Emotional experiences and their explanatory role as mediators between childhood trauma and the EDV have yet to be explored quantitatively. The grounded theory paper [14] also focused more exclusively on Anorexia Nervosa; given research previously has indicated that the EDV and emotional difficulties are common experiences across ED subtypes, the present study seeks to explore the relationships between traumatic childhood experiences, emotional regulation difficulties, negative beliefs about emotional expression and alexithymia and the EDV across ED subgroups.

*Aims*:

- To explore the relationship between traumatic childhood experiences and the eating disorder voice.
- To identify the role of beliefs about emotion, alexithymia, and emotion regulation as potential mediators.

*Hypotheses*:

1. Greater frequency and severity of experiences of childhood trauma (as measured by scores on the Childhood Trauma Questionnaire) will be associated with greater reported power of the eating disorder voice (as measured by the omnipotence subscale of the Beliefs About Voices Questionnaire).
2. Emotional regulation difficulties, alexithymia, and negative beliefs about emotional expression will predict perceived eating disorder voice power.
3. The relationship between childhood trauma and the eating disorder voice will be mediated by negative beliefs about emotional expression and greater emotion regulation difficulties.

### Methods

### Participants

Participants were recruited via social media and via a student sample at Cardiff University. The study advert and link to the Qualtrics survey were shared via Twitter, Instagram, and Facebook, using the first author’s professional social media accounts. Relevant accounts, such as other eating disorder researchers, third-sector organisations and those of individuals with lived experience who post openly about their recovery journey were approached via Twitter and Instagram and shared the adverts to increase exposure. Relevant Facebook groups were also approached and asked to share the advert to their page. To reach the student sample, the study was uploaded to the Experimental Management System (EMS), through which students at Cardiff University are able to complete research to earn credits needed for their studies.

To aid with recruitment and to access as diverse a sample as possible, it was agreed that participants would be eligible for the study if they were over 18 years old, and self-reported experience of an eating disorder voice (described in the advert, for the purpose of this study, as *“an internal commentary which focuses on weight*,* shape*,* and diet and how these relate to self-worth. It can be a companion and helpful guide at first to some people*,* but over time can become degrading and punitive*,* and can make recovering from eating disorders difficult.”*). Participants were not required to have a diagnosis of an ED, nor have received any formal treatment for one (to allow for exploration of a wider range of experiences). However, the Eating Disorder Examination Questionnaire (EDE-Q) was included to explore symptomatology and ED cognitions, along with demographic questions to ascertain diagnoses and treatment histories.

### Procedure

Participants accessed the survey, created using the programme Qualtrics, via a link in the advert as seen on social media, or via the link provided on the Cardiff University student recruitment site. Participants had to read the information sheet and complete the consent form prior to completing the questionnaires. The first page following the consent form explored demographics, including questions regarding age, gender, sexuality, and ethnicity, as well as eating disorder and mental health diagnoses and treatment history. Following completion of demographic information, each questionnaire was presented on a separate page of the survey; the order was randomised, to account for potential order effects. A debrief page was presented at the end of the survey, or if participants chose to withdraw at any point in the survey (by selecting “I wish to withdraw”, an option presented at the bottom of each survey page). The survey took around 20 min to complete, in total.

To compensate for their time, participants were able to provide their email address to enter a prize draw to win a £50 shopping voucher, if they chose. All participants recruited via the student recruitment site were awarded credits for their engagement.

### Measures

*Childhood trauma questionnaire (CTQ)* [36].

A 28-item questionnaire measuring traumatic childhood experiences- participants rate statements about childhood experiences on a 5-point likert scale. It produces 5 subscales- emotional abuse, sexual abuse, physical abuse, emotional neglect, and physical neglect. The CTQ has demonstrated good psychometric properties in eating disorder populations [10]. For the current study, all of the subscales demonstrated good internal consistency (emotional abuse Cronbach’s *α* = 0.91; Physical abuse *α = 0.91;* sexual abuse *α* = 0.95; emotional neglect *α = 0.94;* Physical neglect *α =* 0.82).

Example question: “*When I was growing up… people in my family said hurtful or insulting things to me”: Never true*,* rarely true*,* sometimes true*,* often true*,* very often true*.

*Beliefs about emotions questionnaire (BAEQ)*[37].

This scale measures beliefs about how acceptable it is to experience and express different emotions. It consists of 12 items, scored on a 7-point likert scale. The current study found good internal consistency in scores on the BAEQ (*α* = 0.92).

Example question: “*It is a sign of weakness if I have miserable thoughts”: Totally agree*,* Agree very much*,* Agree slightly*,* Neutral*,* Disagree slightly*,* Disagree very much*,* Totally disagree.*

*Difficulties in Emotion Regulation Scale (brief version- DERS-16)* [38].

This scale measures difficulties in regulating emotions, specifically; nonacceptance of negative emotions (three items), inability to engage in goal-directed behaviours when distressed (three items), difficulties controlling impulsive behaviours when distressed (three items), limited access to emotion regulation strategies perceived as effective (five items), and lack of emotional clarity (two items). The DERS-16 [38] is a short-form version of the 36-item DERS [39]. Reliability and validity of the short-form scale in clinical and non-clinical samples has been explored [38]; they found that scores on the DERS-16 correlated significantly with the DERS. The short form was selected for the current study to reduce the time it takes participants to complete the battery of tests. The current study found good internal consistency for the DERS-16, with an *α* of 0.9.

Example question: *“When I am upset*,* I feel out of control”- Almost never: 0–10%*,* Sometimes: 11–35%*,* About half the time: 36–65%*,* Most of the time: 66–90%*,* Almost always: 91–100%*.

*Beliefs About Voices Questionnaire (BAVQ)* [40].

This is a 35-item questionnaire which explores appraisals of voice characteristics (voice benevolence, malevolence, and omnipotence), and engagement with and behavioural responses to the voice(s). Only responses related to appraisals of the voice (specifically, omnipotence) were utilised in the analysis for the present study. This scale was adapted to specify the eating disorder voice, instead of “voices”, for the purpose of this study. The BAVQ subscales of malevolence, benevolence and omnipotence demonstrated satisfactory internal consistency within the present study (Malevolence *α* = 0.77; benevolence *α* = 0.79; omnipotence *α* = 0.78).

Example question: *“My voice is very powerful” (Disagree*,* Unsure*,* Slightly Agree*,* Strongly Agree).*

*Toronto Alexithymia Scale (TAS-20)* [41, 42].

This is a 20-item questionnaire exploring Alexithymia. It can produce 3 subscales: identifying feelings, communicating feelings, and external thinking, as well as a total score. It is the third iteration of the TAS. The current study identified good internal consistency for the TAS (*α* = 0.87).

Example question: *“I am often confused about what emotion I am feeling”: 1- Strongly disagree*,* 2- disagree*,* 3- Neither agree nor disagree*,* 4- agree*,* 5- strongly agree.*

*Eating Disorders Examination Questionnaire (EDE-Q*,* version 6)-* [43].

The EDE-Q is a 28-item measure of eating pathology. It consists of four subscales measuring cognitions: weight concern, shape concern, eating concern and dietary restriction. The frequencies of eating disorder behaviours over the past 28 days are also measured. The EDE-Q has demonstrated good psychometric properties in eating disorder samples [44, 45]. The EDE-Q Global scale demonstrated good internal consistency (*α* = 0.95), calculated across the 22 item-level indicators contributing to the four EDE-Q subscales.

Example question: *“On how many of the past 28 days… Have you been deliberately trying to limit the amount of food you eat to influence your shape or weight (whether or not you have succeeded)?”: NO DAYS-0*,* 1–5 DAYS-1*,* 6–12 DAYS-2*,* 13–15 DAYS-3*,* 16–22 DAYS-4*,* 23–27 DAYS-5*,* EVERY DAY-6.*

### Ethics

Full ethical approval to conduct the current study was granted by the Cardiff University School of Psychology Ethics Committee prior to recruitment (reference number: EC.23.04.25.6786R). Participant safety was given considerable thought, as the psychological impact of answering questionnaires relating to trauma, emotions and eating disorders has the potential to be high. To ensure informed consent, the information sheet included clear information regarding the nature of the questionnaires, as well as recommendations for participants to ensure they consider their own wellbeing by, for example, having someone around they can seek support from when completing the questionnaires, if needed. A comprehensive consent form outlining right to withdraw, potential harm and benefits of completing the study and data management was also included. A debrief page was included at the end, with support links and phone numbers as well as two calming YouTube videos they could watch if they chose. Researchers’ contact information was also provided, if participants had any questions or concerns following completion of the study. Participants completing the survey via the Cardiff University student recruitment site had a separate debrief page, with additional Cardiff University student support information. Additionally, to ensure those who chose to withdraw were able to access the debrief page for support if needed, at the bottom of each page of the survey there was an “I wish to withdraw” option, which, when selected, would automatically end the survey, and take the participant to the debrief page.

Two service user representatives, one of whom had lived experience of an eating disorder, were involved in the initial development of the survey, including writing of the information sheet, consent form and debrief form. Changes were made based on their feedback, with alterations to language suggested to aid comprehension and improve clarity.

### Data analysis

Correlation analysis exploring relationships between each variable were conducted. The first two hypotheses, investigating whether childhood trauma (as measured by the Childhood Trauma Questionnaire) and emotion difficulties (as measured by the Toronto Alexithymia Scale, the Difficulties in Emotion Regulation Scale and the Beliefs about Emotions Questionnaire) predicted appraisals of EDV power (as measured by the Beliefs About Voices Questionnaire omnipotence subscale), were explored using multiple regression analyses, with simultaneous entry. The third hypothesis (interested in the mediating role of emotions in the relationship between childhood trauma and the EDV) was tested using a mediation model within the multiple regression framework. Hayes’s PROCESS macro [46] in SPSS was utilised to conduct the mediation analysis, using model 4 (the mediation model). The datasets generated during the current study are not publicly available but can be sought from the corresponding author upon reasonable request.

## Results

In total, the survey received 279 responses: 170 via social media, 109 via the student recruitment page. Sixty of these respondents had not completed any of the survey beyond the consent form, and thus were automatically excluded, leaving 219 cases. Missing data analyses indicated that a complete case analysis would result in losing 71 cases, 32.42% of the sample. Little’s [48] test for data Missing Completely at Random (MCAR) was non-significant, χ²(2292) = 2258.706, *p* = .686, indicating no evidence of systematic patterns in missingness that would bias complete-case analyses. Although multiple imputation represents a valid alternative under MCAR assumptions and would have permitted inclusion of all cases with partial data, we opted for a complete-case approach to prioritise analytical transparency in this exploratory study. This decision was informed by the use of bootstrapped mediation models, which require careful alignment between imputation and analysis models, and by a priori power analyses indicating that the final analytic sample (*N* = 148) remained adequately powered for the primary analyses.

The final sample included 148 participants: 76 from social media, and 72 from the student recruitment page. A-priori power analyses, produced with G*Power [47] indicated a sample of 138 or more was required for adequate power to detect a significant finding in a multiple regression model with 5 predictors, with 80% likelihood of detecting a significant finding if it exists (*p*<.05) and a medium effect size. Thus, the sample size was adequately powered for the analyses conducted.

Five participants were missing just one question on the EDE-Q each. As indicated in the scoring guide for the EDE-Q [48] subscales and global scores can still be calculated if data are missing; the mean calculated by adding what data was collected and dividing by the number of completed items. It was therefore decided these participants would be included in the analysis, with their mean scores calculated thus.

### Participant demographics

Participants were aged 18–56 years, the mean age 23.68 (SD = 7.90). Participants’ gender identity, sexual orientation and ethnicity were also collected (see Table 1).

**Table 1:** Participant demographics

		*N*	%
Gender	Female	132	89.2%
	Male	2	1.4%
	Non-binary	10	6.8%
	Trans male	3	2.0%
	Trans female	0	–
	Prefer not to say	0	–
	Other	1	0.7%
Sexual orientation	Heterosexual	88	59.5%
	Bisexual	30	20.3%
	Gay/ homosexual	11	7.4%
	Asexual	6	4.1%
	Pansexual	5	3.4%
	Prefer not to say	5	3.4%
	Other	3	2.0%
Ethnicity	White	128	86.5%
	Black	0	–
	Asian	11	7.4%
	Mixed ethnicity	7	4.7%
	Other	1	0.7%
	Prefer not to say	1	0.7%

Data on participants’ eating disorder diagnoses, treatment, and other mental health diagnoses and treatment were also recorded (see Table 2). Of those who self-reported diagnoses of an ED, several different diagnoses were reported (see Table 3). Two participants had selected “not sure” for whether they had a diagnosis of an ED but had provided an ED diagnosis in the text box; these diagnoses were included in Table 3 (one reported Bulimia Nervosa, the other OSFED). One participant selected “yes” for a diagnosis but had written the year of diagnosis instead of the diagnosis received. Therefore, there are 75 participants with listed diagnoses included in Table 3.

Other diagnoses reported by participants included anxiety, generalised anxiety, agoraphobia, social anxiety, emetophobia, obsessive-compulsive disorder (OCD), post-traumatic-stress disorder (PTSD) and complex PTSD, depression, major depressive disorder (MDD), emotionally unstable personality disorder (EUPD), Bipolar disorder, Autism Spectrum Condition (ASC) and Attention-Deficit Hyperactive Disorder (ADHD).

**Table 2:** Participant Eating Disorder and other mental health diagnoses and treatment

		N	%
Eating disorder diagnosed	Yes	74	50%
	No	64	43.2%
	Not sure	10	6.8%
	Yes, in the NHS	24	16.2%
	Yes, privately	11	7.4%
Currently undergoing treatment for an ED	No	113	76.4%
	Yes, in the NHS	54	36.7%
	Yes, privately	19	12.9%
Previous treatment for an ED	No	74	50.3%
Other mental health diagnoses	Yes	77	52.0%
	No	56	37.8%
	Not sure	15	10.1%
Current or previous treatment for other mental health diagnoses	Yes, in the NHS	52	35.1%
	Yes, privately	26	17.6%
	No	70	47.3%

**Table 3:** Diagnoses reported by participants.

Diagnosis	*N*	%
Anorexia Nervosa	48	64%
Bulimia Nervosa	3	4%
Anorexia Nervosa- Restrictive and binge-purge subtypes	1	1.3%
Anorexia Nervosa- restrictive subtype	1	1.3%
Anorexia Nervosa and Bulimia Nervosa	4	5.33%
Atypical Anorexia Nervosa	5	6.67%
EDNOS/ OSFED	3	4%
ARFID	2	2.67%
Anorexia Nervosa and atypical Bulimia Nervosa	1	1.3%
Atypical Anorexia Nervosa and Bulimia Nervosa	1	1.3%
Body Dysmorphic Disorder and emerging Anorexia Nervosa	1	1.3%
Anorexia Nervosa, restricting, atypical Anorexia Nervosa	1	1.3%
Atypical Bulimia Nervosa, Binge-Eating Disorder	1	1.3%
Anorexia Nervosa, and then atypical Anorexia Nervosa	1	1.3%
OSFED and Bulimia Nervosa	1	1.3%
Restrictive Eating Disorder	1	1.3%

Analyses comparing differences between participants recruited from social media and the student recruitment page indicated significant difference in EDE-Q global score *t*(146) = 3.033, *p*=.003. There were also significant differences between the social media and student participants for the BAVQ omnipotence scale (*t*(146) = 5.341, *p*<.001); as well as the CTQ scores for emotional abuse (*t*(146) = 2.517, *p*<.01); emotional neglect (*t*(146) = 4.713, *p*,0.001); physical neglect (*t*(146) = 2.285, *p*=.02)

**Table 4:** Means and standard deviations of social media and student samples

Questionnaire	Social media sample mean(SD)	Student sample mean(SD)
EDEQ Global	3.77(1.39)	3.05(1.53)
BAVQ	16.78(3.82)	13.50(3.62)
CTQ- Emotional abuse subscale	14.08(6.13)	11.65(5.57)
CTQ- Emotional neglect subscale	14.39(5.11)	10.43(5.12)
CTQ- Physical neglect subscale	9.08(3.92)	7.65(3.66)
CTQ- Sexual abuse subscale	8.71(6.13)	7.46(5.30)
CTQ- Physical abuse subscale	7.53(4.62)	6.58(3.63)
BAEQ	34.67(13.62)	35.81(13.03)
DERS	56.39(12.36)	55.10(11.87)
TAS	59.51(12.97)	58.51(13.07)

*EDE-Q scores*.

A majority (67.57%) of participants scored above clinical cut off (2.8) on the EDE-Q global score. The overall mean score for each EDE-Q subscale and the global score was above clinical cut off, with the exception of the eating concern subscale, which was just below (see Table 4 for details).

### Correlations

Table 5 depicts how each variable correlates, with use of Pearson’s correlation, as data met the required assumptions for parametric testing.

### Relationship between childhood trauma and the eating disorder voice

A simultaneous linear regression was conducted to explore the relationships between different forms of childhood trauma and eating disorder voice omnipotence (as a measure of voice power), whilst controlling for the other forms of abuse. Data met the required assumptions for multiple regression. There was independence of residuals, as assessed by a Durbin-Watson statistic of 1.787. Homoscedasticity and linearity were confirmed, as assessed by visual inspection of a plot of studentised residuals versus unstandardised predicted values. No data points appeared to have excessive influence, as measured by Cook’s Distance, nor did any data point appear to be an outlier, or to have high leverage points. Residuals also appeared to be approximately normally distributed, as indicated by the P-P plots.

Initially, all the subscales of the childhood trauma questionnaire (emotional, sexual, and physical abuse, and emotional and physical neglect) were entered into the model simultaneously. Although variance inflation factor (VIF) and tolerance statistics did not exceed conventional thresholds, emotional abuse showed strong bivariate correlations with both emotional neglect and physical neglect (r’s > 0.70), indicating substantial shared variance and raising concerns about the stability and interpretability of individual regression coefficients.

Given this overlap, and consistent with previous EDV research emphasising abuse-related trauma, a secondary model was estimated excluding emotional and physical neglect to examine whether abuse subscales demonstrated clearer unique associations with voice omnipotence. In the full model including all CTQ subscales, no individual predictor reached statistical significance. In the reduced model excluding neglect subscales, emotional abuse emerged as a significant predictor of voice omnipotence while controlling for physical and sexual abuse, *t*(144) = 2.155, *p* = .033. Physical abuse (*t*(144) = − 0.750, *p* = .454) and sexual abuse (*t*(144) = 0.083, *p* = .934) remained non-significant.

**Table 5:** Correlation matrix

	M	SD	1	2	3	4	5	6	7	8	9	10	11	12	13	14
1. CTQ emotional abuse	12.90	6.00	-													
2. CTQ physical abuse	7.07	4.18	0.615**	-												
3. CTQ sexual abuse	8.10	5.76	0.424**	0.539**	-											
4. CTQ emotional neglect	12.47	5.47	0.731**	0.504**	0.331**	-										
5. CTQ physical neglect	8.39	3.90	0.725**	0.662**	0.443**	0.691**	-									
6. TAS	59.03	12.98	0.265**	0.197*	0.202*	0.216**	0.274**	-								
7. DERS	55.76	13.00	0.252**	0.138	0.154	0.157	0.141	0.540**	-							
8. BAEQ	60.80	13.31	0.267**	0.175*	0.137	0.216**	0.214**	0.516**	0.542**	-						
9 BAVQ- omnipotence	15.18	4.06	0.177*	0.059	0.058	0.166*	0.153	0.187*	0.370**	0.279**	-					
11. EDEQ- restraint	2.92	1.83	0.152	0.148	0.093	0.239**	0.217**	0.277**	0.304**	0.287**	0.446**	0.533**	-			
12. EDEQ- eating concern	2.68	1.63	0.308**	0.217**	0.143	0.291**	0.276**	0.412**	0.385**	0.314**	0.470**	0.534**	0.760**	-		
13. EDEQ- shape concern	4.19	1.52	0.332**	0.222**	0.188*	0.301**	0.266**	0.354**	0.389**	0.331**	0.424**	0.506**	0.737**	0.730**	-	
14. EDEQ- Weight concern	3.90	1.64	0.308**	0.235**	0.213**	0.293**	0.250**	0.289**	0.302**	0.304**	0.425**	0.469**	0.731**	0.724**	0.897**	-
15. EDEQ- Global	3.42	1.50	0.298**	0.225**	0.173*	0.308**	0.277**	0.365**	0.379**	0.339**	0.487**	0.564**	0.898**	0.886**	0.922**	0.920**

*= p<.05

**= p<.01.

### Relationship between emotion regulation and the eating disorder voice

A simultaneous linear regression was conducted to explore the relationships between difficulties in emotion regulation, alexithymia, and beliefs about emotions and eating disorder voice omnipotence (as a measure of voice power), whilst controlling for the other emotional difficulty measures. Again, the data met the required assumptions for multiple regression.

Alexithymia, beliefs about emotions, and emotion regulation statistically significantly predicted voice omnipotence: *F*(3, 144) = 8.325, *p*<.001. However, R² for the overall model was 14.8% and adjusted R² was 13%, indicating only a small effect.

Emotion regulation was the only significant predictor of voice omnipotence when controlling for the other emotion measurements: *t*(144) = 3.372, *p*<.001. Alexithymia did not predict voice omnipotence: *t*(144)=-0.613, *p*=.541; nor did beliefs about emotions: *t*(144)= -1.351, *p*=.179.

### Mediating role of emotions

As emotional abuse was established as a significant predictor of voice omnipotence (both on previous research and the regression analyses of the present study), further analysis was conducted to explore the potential mediating role of emotions.

Hayes’s [46] PROCESS macro via bootstrapping method was utilised to explore emotion regulation, alexithymia, and beliefs about emotion as mediators between childhood emotional abuse and voice omnipotence (indirect effect = path a x path b, a = the effect of childhood trauma on emotion regulation, alexithymia, and beliefs about emotion, b = the effect of emotion regulation, alexithymia, and beliefs about emotion on voice omnipotence). The indirect effect is only statistically significant if its bias corrected 95% confidence intervals (CI) around the indirect effect from 5000 bootstrap re-samples excluded zero.

**Fig. 1:**
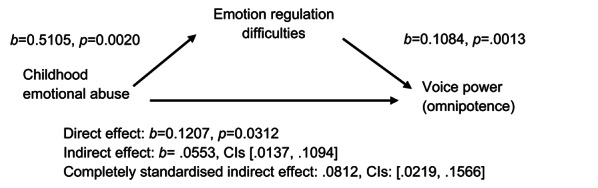
Mediation model for childhood emotional abuse, emotion regulation difficulties and voice omnipotence

The findings of the mediation analyses indicated that only scores on the DERS, that is, emotion regulation, was a significant mediator for childhood emotional abuse and voice omnipotence. There was a significant total effect between childhood emotional abuse and voice omnipotence (*b* = 0.1207, *p* = .0312), and path *a* (i.e., emotional abuse on emotion regulation difficulties) (*b* = 0.5105, *p* = .0020) and path *b* (i.e., emotion regulation difficulties on voice omnipotence) (*b* = 0.1084, *p *= .0013) were both significant. When emotion regulation entered the relationship between childhood emotional abuse and voice omnipotence, the effect was significant (*b *= 0.0812, CIs: [0.0219, 0.1566]). (See Fig. 1 for mediation model.)

## Discussion

### Summary of findings

The findings of the present study partially support the hypotheses proposed: childhood trauma (specifically, emotional abuse experiences) significantly predicted voice power (as measured by the voice omnipotence subscale of the BAVQ), and emotion regulation was a partial mediator for this. Emotion regulation, but not beliefs about emotion or alexithymia, significantly predicted higher ratings of voice omnipotence, although only a small effect was observed. Voice omnipotence was also significantly positively correlated with all subscales (including global) of the EDE-Q. Whilst directionality cannot be determined, these correlations align with the findings of Pugh and colleagues [10], which also identify an association between voice power and ED pathology, and extends their work by exploring a differing mediator. The previous study [10] focused on dissociation as a key mediator, supporting the trauma-dissociation model [49], which posits that internal voices represent traumatic content from maltreatment, the processing of which was disrupted by a dissociative process that causes the voices to be experienced as external/ separate as opposed to memory. The present study indicates emotion regulation difficulties likely also play a significant mediating role. A recent article [8] discussed several models of the eating disorder voice (EDV), including the interpersonal theory of voices. This model proposes that the ways in which individuals relate to and interact with the EDV are particularly important and often mirror patterns present in their relationships with others. For example, individuals who have been positioned as victims or have adopted submissive roles in interpersonal relationships may experience similar dynamics in their voice-hearing experiences, perceiving the EDV as more powerful and domineering while adopting a more submissive stance. The unique importance of emotional abuse in predicting the perceived power of the EDV in the present study provides some support for this model and suggests that further investigation into relationship dynamics may help to clarify how the EDV develops and is experienced. Emotion regulation, alongside other variables, may act as mediators within this; emotions shaping how we respond to and manage difficulties and relate to ourselves and others [29]. This is of particular significance, given some research has found that patterns of submitting to powerful EDVs can worsen ED pathology [50] and working on healthier interactions with the EDV can be of use in treatment [7, 19].

While the current study findings present several limitations and thus should be interpreted with caution, the findings of significant links between childhood emotional abuse and EDV power add indicate some support for the theory that childhood adversity may be linked to voice-related experiences within ED populations [10, 14]. The findings also more broadly expand on previous research indicating ED pathology (in this case, the EDV) is associated with childhood adversity through the mediating influence of emotion regulation difficulties [28, 29, 32, 33, 34].

The present study provides some evidence in support of the theorised role of emotionally invalidating environments (which is not unique to emotional abuse but is most commonly associated with this form of abuse) in ED pathology [25, 26] and could indicate that a mechanism through which emotionally invalidating environments and associated emotion regulation difficulties impact ED pathology is the EDV. As theorised in a prior qualitative study [14] the EDV may play a unique role in offering (conditional) safety, support, and comfort during periods of relational and internal unsafety, and helping to manage the associated emotional difficulties. The findings of the present study provide preliminary support for this theory; given the significant relationships between emotional abuse, emotion regulation difficulties, and EDV power, as well as the significant positive correlations between these factors and EDE-Q scores. Although no directional conclusions can be drawn; further investigation of the unique significance of the EDV is indicated based on the current findings.

Childhood emotional abuse (CEA) specifically was the only CTQ subscale which was directly associated with the EDV. This echoes findings of other research [10], which identified that dissociation partially mediated the relationship between CEA and the EDV, but no other forms of abuse. There are a few possible reasons for this; firstly, CEA has been found to be of particular relevance to ED pathology [51, 52], and may be of unique relevance to the EDV specifically, based on qualitative research indicating the EDV can mimic patterns of emotional abuse in its interactions with individuals [12, 13, 14]. Additionally, research has found that the mechanisms through which different forms of trauma influence ED pathology appear to differ across types of traumas. For example, an individual’s relationship with their body may be of particular importance in childhood sexual abuse [53]. Thus, the EDV and its emotion regulation functions may be more particularly relevant to CEA, with other mechanisms at work in other forms of abuse. These findings imply that the EDV may be more uniquely attributed to the impacts of CEA and emotional invalidation (which can be experienced through all forms of abuse [25]). These findings are only preliminary, however, and indicate further investigation of the relationships between childhood trauma and the EDV experience could be important in developing an understanding of this commonly experienced feature of EDs.

While emotion regulation significantly predicted EDV omnipotence and was a significant mediator between CEA and EDV omnipotence, beliefs about emotion and alexithymia were not found to be significant predictors or mediators. There are several potential explanations for this. Firstly, it is possible that emotion regulation is the most important mediator, and so when the analysis was run whilst controlling for emotion regulation, alexithymia and beliefs about emotion were no longer significant. Indeed, when not controlling for other variables, both the alexithymia and beliefs about emotions measurements were significantly positively correlated with voice omnipotence (as reported in the correlation matrix). Based on theory discussed in the introduction [25, 26, 30], this perhaps makes sense; alexithymia and beliefs about emotion may be contributory factors of emotion regulation difficulties, and so are perhaps of less direct relevance to the EDV. Additionally, the inclusion of all eating disorder and disordered eating experiences may have influenced these findings. While all participants had experience of an EDV, a range of eating difficulties were explored, and diagnosis was not a requirement. Evidence suggests that emotion regulation difficulties are common across ED diagnoses and across psychopathology more generally [35], whereas some research indicates nuance in alexithymia and beliefs about emotions across ED subtypes. For example, a review of the literature [54] found variation in conclusions across studies, but found evidence to suggest alexithymia is more directly associated with AN and restrictive eating difficulties, as opposed to binge-purge types. Another study [31] found differences in suppression of emotion between ED subgroups, with those with restrictive presentations significantly more likely to suppress emotion. The authors highlight the potential importance of exploring ED subtypes separately, given the nuance in presentation. Thus, it is possible that alexithymia and beliefs about emotion may vary across ED diagnoses, with emotion regulation a more consistent difficulty across eating pathology. It was not possible in the present study to run the analyses for individuals with AN separately, due to the smaller number of participants in the sample with self-reported diagnosis of AN. However, future research may seek to explore if this mediation model differs within clinical samples of individuals with differing ED diagnoses.

### Limitations and future research

There were several limitations to the present study. Firstly, whilst the included sample was adequately large, it lacked diversity and included predominantly younger, white, cisgender females. The current sample especially is lacking in representation of different genders and ethnic minorities; not an uncommon problem in ED research, and something that requires addressing [55]. Future research into the EDV could benefit from examining this phenomenon in more diverse populations than the present study.

Secondly, it was beyond the scope of the present study to collect data from a clinical sample; only a community sample was surveyed. Only 50% of the participants self-reported an ED diagnosis, and 23.6% had accessed treatment for an ED, past or present. Mean scores for the EDE-Q, however, indicated that a majority of participants were above the recommended clinical cut off of 2.8 [56]. The present study findings suggest further analysis, within a clinical sample, could be warranted. In addition, due to missing data, a large proportion of data was removed; while Little’s [48] test of data Missing Completely at Random suggests there was no pattern to who completed the survey and who did not, this is of importance to consider, as a lot of experiences were potentially missed. Samples recruited through social media and through Cardiff University also differed significantly in scores on all measures included: the sample, therefore, is quite heterogeneous. Additionally, the inclusion of multiple statistical tests increases the risk of Type 1 error.

Thirdly, the only measure of childhood trauma utilised was the Childhood Trauma Questionnaire, which focuses specifically on child abuse experiences within the family. Two potentially relevant predictors (emotional and physical neglect) were also excluded from the analysis, due to possible multicollinearity effects; meaning the present study was unable to explore these potentially highly relevant trauma experiences. Participants in a recent qualitative study [14] discussed significance of traumatic experiences outside of abuse, such as traumatic school and peer-related experiences, in relation to the eating disorder voice; thus, future research could benefit from including more comprehensive examinations of childhood trauma experiences. Future research may also seek to develop and evaluate tools to measure the EDV more specifically; the current study utilised voice-hearing questionnaires, with the wording adapted. Development of more specific tools for measuring EDV characteristics could be beneficial for furthering research into this phenomenon and assessing voice experiences clinically.

Additionally, no conclusions regarding causality can be drawn, given the cross-sectional nature of the study. Directionality is also not possible to establish; in order to examine this further, longitudinal investigations are required. The results and interpretations of findings should be considered carefully within this context.

Other factors likely play a role in aetiology of the EDV. A recent grounded theory paper [14] explored experiences of unsafety beyond difficulty with emotion; for example, the importance of identity and sense of self within this population. Another study [30] outlines how emotional difficulties can result in a ‘lost sense of emotional self’, with AN becoming a way of both managing emotion difficulties and providing a ‘false sense of self’ [57]. The grounded theory study [14] found that participants reported the Anorexic Voice provided them with an acceptable identity, countering the belief that they are a bad person. The role of identity in the EDV could be important to investigate in future research. Additionally, a meta-analytic review [58] suggested perfectionism and emotion dysregulation were two mediating factors between insecure attachment and AN. Morrison and colleagues [14] found that the voice can adopt perfectionistic standards, and proposes that the voice may be a mediator in the relationship between perfectionism and AN. Identity, perfectionism and associated sense of self-worth, while beyond the scope of the present study, may be important factors, alongside emotion regulation, in the EDV; future research could seek to explore this further.

### Clinical implications

The present study’s findings are consistent with other research indicating relevance of childhood trauma to emotion regulation difficulties and eating disorder pathology. As such, screening for trauma experiences, in particular emotional abuse, could be indicated; in particular when an individual reports experience of an EDV.

In addition, the findings support the importance of consideration of emotion regulation in the conceptualisation and treatment of EDs. Emotion regulation difficulties may be particularly important to explore for individuals who report a history of childhood emotional abuse and/or emotionally invalidating environments [25] and could be important to address in treatment. The current research supports this and also indicates further exploration of emotion regulation may be beneficial when individuals report a voice, in particular when that voice is experienced as very powerful.

Clinicians would benefit from considering the challenges of engaging in treatment which might directly contradict the EDV, as well as the potential emotional impact of resisting the voice when it perhaps provides important emotion regulation functions. The EDV could be a way of managing the emotions associated with past traumatic experiences, and clients may need support in processing past trauma and effectively regulating emotions in other ways, for more effective treatment outcomes.

## Conclusion

The present study has found evidence in support of the association between childhood emotional abuse and the eating disorder voice experience, and for the mediating role emotion regulation plays in this relationship. The findings could be interpreted to indicate an important role of the EDV within the already-established relationship between childhood emotional abuse, emotion regulation difficulties, and ED pathology more broadly. Further research is required to better understand the unique role of the EDV; the present study adds to the growing evidence base supporting its relevance in understanding, working with, and treating eating disorders.

## Data Availability

The datasets generated during the current study are not publicly available but can be sought from the corresponding author upon reasonable request.
